# The effect of daytime napping and full‐night sleep on the consolidation of declarative and procedural information

**DOI:** 10.1111/jsr.12649

**Published:** 2017-12-22

**Authors:** Frank J. van Schalkwijk, Cornelia Sauter, Kerstin Hoedlmoser, Dominik P. J. Heib, Gerhard Klösch, Doris Moser, Georg Gruber, Peter Anderer, Josef Zeitlhofer, Manuel Schabus

**Affiliations:** ^1^ Laboratory for Sleep, Cognition and Consciousness Research Centre for Cognitive Neuroscience (CCNS) University of Salzburg Salzburg Austria; ^2^ Department of Neurology Medical University of Vienna Vienna Austria; ^3^ Competence Center of Sleep Medicine Charité – University Medicine Berlin Germany; ^4^ Department of Psychiatry and Psychotherapy Medical University of Vienna Vienna Austria

**Keywords:** memory, mirror‐tracing, polysomnography, spindles, word‐pair learning

## Abstract

Many studies investigating sleep and memory consolidation have evaluated full‐night sleep rather than alternative sleep periods such as daytime naps. This multi‐centre study followed up on, and was compared with, an earlier full‐night study (Schabus *et al*., 2004) investigating the relevance of daytime naps for the consolidation of declarative and procedural memory. Seventy‐six participants were randomly assigned to a nap or wake group, and performed a declarative word‐pair association or procedural mirror‐tracing task. Performance changes from before to after a 90‐min retention interval filled with sleep or quiet wakefulness were evaluated between groups. Associations between performance changes, sleep architecture, spindles, and slow oscillations were investigated. For the declarative task we observed a trend towards stronger forgetting across a wake period compared with a nap period, and a trend towards memory increase over the full‐night. For the procedural task, accuracy was significantly decreased following daytime wakefulness, showed a trend to increase with a daytime nap, and significantly increased across full‐night sleep. For the nap protocol, neither sleep stages, spindles, nor slow oscillations predicted performance changes. A direct comparison of day and nighttime sleep revealed that daytime naps are characterized by significantly lower spindle density, but higher spindle activity and amplitude compared with full‐night sleep. In summary, data indicate that daytime naps protect procedural memories from deterioration, whereas full‐night sleep improves performance. Given behavioural and physiological differences between day and nighttime sleep, future studies should try to characterize potential differential effects of full‐night and daytime sleep with regard to sleep‐dependent memory consolidation.

## Introduction

Sleep after learning contributes to memory consolidation, which is expressed by significantly better memory performance as compared with a similar period of wakefulness (Diekelmann and Born, 2010; Walker *et al*., 2002). Specifically, sleep contributes to the consolidation of procedural (Plihal and Born, 1997; Stickgold *et al*., 2000; Walker *et al*., 2003) and declarative memories (Plihal and Born, 1997; Schabus *et al*., 2004; Tucker *et al*., 2006). Whereas the classical view relates slow‐wave sleep (SWS) to the consolidation of declarative information and rapid eye movement (REM) sleep to the consolidation of procedural information (Plihal and Born, 1997; Wagner *et al*., 2003), many other studies could not find such associations at this macro‐level (for review, see Ackermann and Rasch, 2014; Stickgold, 2013). Furthermore, it has been suggested that to be consolidated, specific memories need to be processed on different levels, with for example gross‐motor learning and fine‐motor tuning relying on different sleep mechanisms, such as REM sleep and sleep spindles, respectively (Smith *et al*., 2004).

On a more detailed level, non‐REM (NREM) spindles and slow waves are believed to play important roles for memory consolidation, with spindles being positively associated with declarative learning (Gais *et al*., 2002), declarative overnight performance changes (Clemens *et al*., 2005; Schabus *et al*., 2004) and intelligence (Bódizs *et al*., 2014; Fogel *et al*., 2007; Schabus *et al*., 2006; Ujma *et al*., 2014). In addition, slow‐wave activity (Holz *et al*., 2012), amplitude, and the length of the up‐state have been positively related to overnight memory consolidation (Heib *et al*., 2013).

The majority of early sleep studies have focused on the relevance of full‐night sleep for memory consolidation and performance. Yet, for practical reasons many scientists seem to have changed their focus to shorter sleep durations; especially daytime naps. Several nap studies have indicated that also daytime sleep positively affects consolidation of declarative (Schabus *et al*., 2005; Schmidt *et al*., 2006; Tucker *et al*., 2006; Van Der Helm *et al*., 2011) and procedural information (Backhaus and Junghanns, 2006; Cajochen *et al*., 2004; Nishida and Walker, 2007). Yet, these positive effects of a daytime nap on memory performance are not always found for both memory modalities (e.g. significant effects for declarative but not procedural tasks; Tucker *et al*., 2006; Wilhelm *et al*., 2008). Therefore, the effects of sleep on procedural and declarative memory should be studied using identical laboratory settings and learning task specifications, and ideally directly compare the effects of short daytime versus full‐night sleep.

While nap studies often allow for 1–3 h nap opportunities, there is a study (Lahl *et al*., 2008) that reports that short (35.8 ± 8.9 min) and even very short sleep periods (6.3 ± 1.7 min) can result in significantly higher word‐pair recall performance (as compared with wake). Another study compared varying durations of night sleep (3.5 h versus 7.5 h) and found similar overnight improvements on word‐pair association and procedural finger‐tapping (Tucker and Fishbein, 2009). Given this evidence, daytime naps and full‐night sleep seem to be considered similar in their behavioural outcomes and are therefore often used interchangeable when sleep‐dependent memory consolidation is discussed.

However, studies directly contrasting naps and full‐night sleep are rare and have casted a less clear picture. While comparable effects are, for example, reported for a visual texture‐discrimination task (Mednick *et al*., 2003), recall of semantically unrelated word‐pairs was found to be better after full‐night sleep (Lo *et al*., 2014). Besides the obvious fundamental differences between daytime naps and full‐night sleep, which are the sleep duration and the timing of sleep, other aspects should be considered. In day‐ and nighttime studies the time of learning and recall is, for example, shifted. Given the considerable effects of time awake (i.e. the sleep homeostasis) and circadian variations on cognitive performance (Schmidt *et al*., 2007) and brain activity (Muto *et al*., 2016), it therefore seems highly warranted to start evaluating how far daytime napping and nighttime sleep are really comparable. This study aimed to critically test the effects of a daytime nap on consolidation of both declarative and procedural information. In addition, we present our results in comparison to a previous full‐night study where we investigated the effects of full‐night sleep using identical declarative (Schabus *et al*., 2004) and procedural tasks. While this comparison is of an exploratory nature, it is of practical significance, as such comparisons are practically absent in published literature. Our present paper attempts to offer a first contribution in this direction, and intends to raise the awareness that it may be unjustified to expect the same memory benefits and associations with sleep patterns when testing across short daytime as compared with full nighttime sleep.

## Materials and methods

### Participants

Participants were healthy students from the University of Salzburg and Medical University of Vienna, Austria. Written informed consent was obtained prior to participation. Participants were screened for anamnesis or somatic findings, and evaluated on sleep quality [Pittsburgh Sleep Quality Index (PSQI)], intelligence (Raven's Advanced Progressive Matrices), and chronotype [Morningness–Eveningness Questionnaire (D‐MEQ)]. Exclusion criteria were: (1) sleep duration <6 h per night; (2) history of drug abuse or habituation (including alcohol); (3) any requirement of psychoactive medication or other medication that might interfere with study assessment (e.g. beta blockers); (4) night‐shift employees; (5) PSQI global score >5; (6) mean bedtimes <22:00 hours or >00:00 hours; (7) anxiety – Self‐rating Anxiety Score raw scores ≥ 36; (8) depression – Self‐rating Depression Scale raw scores ≥40; and (9) inability or unwillingness to comply with the study protocol. *Post hoc* exclusion criteria included any pathological findings during initial screening or an inability to fall asleep during the nap opportunity. From the initial sample (*N *= 95), participants were excluded due to exclusion criteria (*n *= 1), dropout (*n *= 7), or outlier detection on either behavioural performance or sleep architecture (M ± 3SD; *n *= 11). The final analysed sample consisted of 76 participants (M_age_ = 23.34 years, SD = 2.40, range = 20–30 years; 52.6% male). D‐MEQ scores revealed that most participants were indifferent regarding their morningness/eveningness preference (52.07 ± 10.04; range = 32–76), with only four participants being definitive morning types (*N*_nap _= 2). To contrast the effects of a nap and full‐night sleep on declarative and procedural memory, data from a previous study were added that evaluated 48 participants using identical paradigms (Schabus *et al*., 2004). Sleep‐related effects on declarative memory (*n *= 24) have already been published elsewhere (Schabus *et al*., 2004). After excluding outliers based on behavioural performance (M ± 3SD; *n *= 1), long sleep‐onset latency (>20 min; *n *= 2), and low sleep efficiency (<80%; *n *= 1), analyses for the procedural task were conducted on 20 participants (M_age_ = 23.40 years, SD = 2.78; range = 20–30 years; 45% male). Due to technical difficulties, mirror‐tracing speed could only be analysed for 14 participants.

### Experimental paradigm

Participants wore an actigraph on the non‐dominant wrist and maintained a daily sleep diary throughout the paradigm (14 days). The first recording was scheduled during the first week of the study. Initial briefing and electroencephalographic (EEG) preparations were conducted in the morning (09:00–11:00 hours). Participants were randomly assigned to either the nap or wake group, and were further assigned to the declarative (*N *= 40; *n*_nap_ = 18; *n*_wake_ = 22) or procedural task (*N *= 36; *n*_nap_ = 17; *n*_wake_ = 19). Condition order (starting with learning or control condition) was randomized. The first recording session (11:00–13:00 hours) included acquisition and immediate retrieval (retrieval 1) of the relevant memory task or relative control task. One hour after completing the immediate retrieval a retention period of 90 min (14:00–15:30 hours) provided participants either with a nap opportunity or period of quiet wakefulness in combination with polysomnography (PSG). Participants were informed of their group assignment after completing the first retrieval session. Retention and subsequent delayed retrieval (retrieval 2; 16:30–17:30 hours) were separated by 1 h to account for sleep inertia. The second recording day, scheduled during the second week of the study, repeated this protocol. Participants conducted either the relevant control or learning task depending on the first session. The protocol was approved by the University Committee.

### Instruments

#### Declarative memory task

A word‐pair task (WPT) of 160 word‐pairs applied an adapted paradigm from Plihal and Born (1997) and previous studies (Schabus *et al*., 2004, 2006). Word‐pairs were presented twice in a blocked and randomized order (Fig. S1a). Retrieval used a cued recall paradigm (Fig. S1b) that rated performance on the percentage of correct retrievals. The control task instructed participants to count and verbally report the number of deviating letters within each pseudo word‐pair (Fig. S1c and d).

#### Procedural memory task

In a mirror‐tracing task (MTT), adapted from Plihal and Born (1997), participants had to trace 12 figures as quickly and accurately as possible within a 90‐s time frame using an electronic stylus (Fig. S2). Direct vision of the figures was prevented; instead allowing vision through a mirror. The control task used an identical paradigm with different figures, yet allowed normal observation of the figures. Performance was evaluated based on speed (distance between start and end points) and the percentage of time that the trace deviated from the marked stimulus line (error time). The present study aimed to evaluate a truly implicit and more complex motor adaptation task as compared with more fine‐grained motor tasks such as the finger‐tapping task, as latter tasks focus more on the fine‐tuning of movements (and may even include explicit memory components).

#### EEG and PSG

Both EEG and PSG applied 21 scalp electrodes in combination with the Neuroscan system (NeuroScan, El Paso, Texas, USA). Electrodes were placed following the international 10/20 system (Fp1, Fpz, Fp2, F7, F3, Fz, F4, F8, T3, C3, Cz, C4, T4, T5, P3, Pz, P4, T6, O1, Oz, O2, and included mastoids A1 and A2). PSG recordings included five additional electro‐oculogram channels, one bipolar electrocardiogram channel, one bipolar submental electromyogram channel, and one bipolar respiratory channel. Signals were filtered (high‐pass = 0.10 Hz; low‐pass = 70 Hz; 50 Hz notch filter) and acquired using a 250 Hz sampling rate.

#### Questionnaires

Participants maintained a sleep diary for the duration of the paradigm. The diary included a self‐rating questionnaire for sleep and awakening quality (SSA), subjective estimates of sleep duration and sleep latency, 100‐mm visual analogue scales (ASES) for mood, drive, affectivity and drowsiness, and inquired for the duration of potential naps. Additionally, participants were asked to fill in a collection of questionnaires that included the ASES and Stanford Sleepiness Scale (SSS; Hoddes *et al*., 1972).

### Analyses

Behavioural performance changes were calculated per memory task (retrieval 2 − retrieval 1 performances). Computer‐assisted sleep scoring was conducted in accordance with standard criteria (Rechtschaffen and Kales, 1968) using the Somnolyzer 24x7 (The Siesta Group, Vienna, Austria). All PSG recordings were reviewed visually epoch by epoch and corrections were applied if necessary. Sleep spindles were automatically detected on channels C3 and C4 following previously described criteria (Anderer *et al*., 2005) that included: (1) amplitude ≥12 *μ*V; (2) duration between 0.3 and 2.0 s; (3) frequency range 11–15 Hz; (4) onset during NREM sleep; and (5) controlling for muscle (30–40 Hz) and alpha (8–12 Hz) artefacts. In accordance with earlier studies (Heib *et al*., 2015; Schabus *et al*., 2006), we used spindle activity (duration × amplitude; SpA) for further analyses. Slow‐wave detection was conducted using previously described criteria (Massimini *et al*., 2004) that included: (1) zero crossings separated by 0.3–1 s; (2) negative peak <−80 *μ*V; and (3) peak‐to‐peak amplitude >140 *μ*V. Detection yielded the number and density (number of slow oscillations/SWS duration) of slow oscillations, as well as the peak‐to‐peak amplitude and length of the positive wave. Spindle and slow‐wave detection were conducted using custom scripts with Matlab 9.0 (R2016a; Natick, MA, USA). Statistical analyses were conducted using IBM SPSS Statistics version 22 (Armonk, NY, USA). Comparisons were conducted between groups and between retrieval sessions using independent‐samples *t*‐tests, paired‐samples *t*‐tests, and the Wilcoxon test, and were corrected for multiple comparisons (using Bonferroni) where necessary. Correlations were conducted using Pearson coefficients and Spearman Rho dependent on data distribution. Estimates of effect sizes and 95% confidence intervals (CI) were calculated using the ‘escalc’ function of the ‘metaphor’ package (Viechtbauer, 2017) and ‘effsize’ package (Torchiano, 2017) for R version 3.3.2.

## Results

### Behavioural performance

Performance changes were compared between the two samples and labs. No group differences were observed for performance changes on either memory task (all *P *> 0.160). Consequently, the samples were merged to create general nap and wake groups.

#### Control condition

Performance changes during the control conditions of both WPT (reporting deviating letters) and MTT (regular figure tracing) were investigated (Fig. S3). The control condition of the WPT evaluated response accuracy and response time. For the nap study, the nap group showed a significant improvement in response accuracy [*Z *= −2.256, *P *= 0.024, *d *= −0.62, 95% CI (−1.11, −0.08)], whereas no change was found for the wake group [*Z *= −0.075, *P *= 0.940, *d *= 0.07, 95% CI (−0.35, 0.48)]. Response times for correct trials showed a significant decrease for the nap group [*t*_16_ = 3.06, *P *= 0.008, *d *= 0.74, 95% CI (0.18, 1.24)] as well as for the no‐nap group [*t*_21_ = 2.76, *P *= 0.012, *d *= 0.59, 95% CI (0.12, 1.02)]. The control condition of the MTT evaluated error time (%). No performance changes were found for the nap group [*Z *= −1.335, *P *= 0.182, *d *= 0.01, 95% CI (−0.47, 0.48)] or for the wake group [*Z *= −0.097, *P *= 0.923, *d *= 0.07, 95% CI (−0.38, 0.52)]. There were no group differences in performance during retrieval 1 [*Z *= −1.027, *P *= 0.305, *d *= −0.03, 95% CI (−0.71, 0.64)], retrieval 2 [*Z *= −0.783, *P *= 0.434, *d *= 0.06, 95% CI (−0.62, 0.74)], or their performance changes [*Z *= −1.541, *P *= 0.123, *d *= 0.07, 95% CI (−0.61, 0.75)].

For the full‐night study, no overnight change in accuracy was found during the WPT control condition [*t*_16_ = 0.99, *P *= 0.34, *d *= −0.25, 95% CI (−0.72, 0.24)]. Reaction times showed a slight non‐significant overnight improvement [*t*_16_ = 1.92, *P *= 0.073, *d *= 0.47, 95% CI (−0.04, 1.40); Schabus *et al*., 2004]. Overnight performance changes for the MTT showed a significant decrease in error time from retrieval 1 (1.27 ± 3.43) to retrieval 2 [0.41 ± 1.08; *Z *= −2.373, *P *= 0.018, *d *= 0.68, 95% CI (0.05, 2.25)].

#### Learning condition

**Figure 1:**
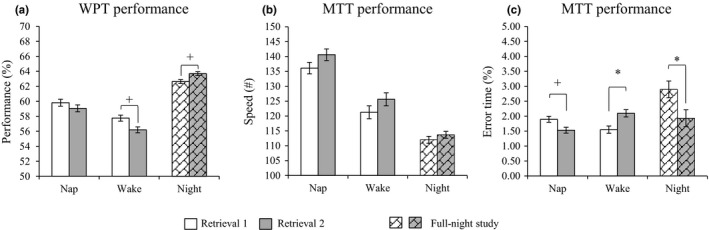
Behavioral performance for both WPT and MTT. Plots illustrate mean ± standard error. Error bars have been adjusted to illustrate within‐subject variability of the repeated‐measures design (for comparison see Fig. S6). For the WPT (a), the night sleep group showed a trending improvement from retrieval 1 to retrieval 2, whereas the wake group showed a trending decrease. For the MTT task (b–c) performance differences from retrieval 1 to retrieval 2 showed a significant increase in error time for the wake group and a decrease in error time for the full‐night sleep group. *P < 0.05,^+^ *P *< 0.10.

Results from the nap study are initially discussed. For the learning condition of the WPT, no group differences were found for retrieval 1 [*t*_38_ = 0.29, *P *= 0.776, *d *= 0.09, 95% CI (−0.55, 0.74)] or for retrieval 2 [*t*_38_ = 0.40, *P *= 0.694, *d *= 0.13, 95% CI (−0.52, 0.77); Fig. 1a]. Relative performance changes from retrieval 1 to retrieval 2 were similar between the nap (−0.75 ± 3.92) and wake (−1.56 ± 3.74) groups [*t*_38_ = 0.67, *P *= 0.506, *d *= 0.21, 95% CI (−0.43, 0.86)]. Performance between retrieval 1 and retrieval 2 was non‐significant for the nap group [*t*_17_ = 0.81, *P *= 0.431, *d *= 0.19, 95% CI (−0.28, 0.65)], whereas the wake group showed a trend towards reduced performance during retrieval 2 [*t*_21_ = 1.96, *P *= 0.063, *d *= 0.42, 95% CI (−0.03, 0.84)]. Similar group comparisons were done for MTT performance changes for speed and error time. For speed (Fig. 1b), there were no group differences on memory performance for retrieval 1 [*t*_34_ = 1.06, *P *= 0.295, *d *= 0.35, 95% CI (−0.33, 1.04)] or retrieval 2 [*t*_34_ = 1.24, *P *= 0.224, *d *= 0.41, 95% CI (−0.27, 1.10)]. Relative performance changes from retrieval 1 to retrieval 2 showed no significant differences between the nap (4.50 ± 16.08) and wake (4.36 ± 18.95) groups [*t*_34_ = 0.02, *P *= 0.981, *d *= 0.01, 95% CI (−0.67, 0.69)]. For error time (Fig. 1c), there were no group differences for memory performance for retrieval 1 [*Z *= −1.190, *P *= 0.234, *d *= 0.24, 95% CI (−0.44, 0.92)] or for retrieval 2 [*Z *= −0.317, *P *= 0.751, *d *= −0.32, 95% CI (−1.01, 0.36)]. Yet, the performance change in error time was significantly different between the nap group (−0.36 ± 0.81) as opposed to the wake group [0.55 ± 1.07; *Z *= −2.524, *P *= 0.012, *d *= −0.95, 95% CI (−1.67, −0.24)]. *Post hoc* tests revealed that for the wake group error time significantly increased [*Z *= −2.027, *P *= 0.043, *d *= −0.51, 95% CI (−0.97, −0.02)], whereas the nap group showed a trending reduction in error time [*Z *= −1.686, *P *= 0.092, *d *= 0.45, 95% CI (−0.07, 0.92)].

Controlling for fatigue showed that the nap group was statistically less alert (SSS retrieval 1: 2.47 ± 1.02; retrieval 2: 2.26 ± 0.71) than the wake control group [retrieval 1: 2.05 ± 0.66; retrieval 2: 1.89 ± 0.65, *F*_1,67_ = 4.671, *P *= 0.034, *d *= 0.51, 95% CI (0.03, 1.00)]. Yet, no overall fatigue was observed in either of the groups (i.e. a value of 2 indicating ‘functioning at high levels but not at peak, and able to concentrate’), nor were there significant changes following either wake or sleep retention intervals [*F*_1,67_ = 1.013, *P *= 0.318, *d *= 0.24, 95% CI (−0.24, 0.71); Fig. S4]. Furthermore, MTT performance was not affected by the condition order (control‐learning versus learning‐control; Fig. S5).

For the overnight study, performance change on the WPT was marginally significant [*t*_23_ = −2.03, *P *= 0.054, *d *= −0.41, 95% CI (−0.81, 0.02); Schabus *et al*., 2004; Fig. 1a]. Performance changes for the MTT revealed no difference in speed between retrieval 1 (111.99 ± 60.14) and retrieval 2 (113.67 ± 64.33), [*t*_13_ = −0.73, *P *= 0.479, *d *= −0.19, 95% CI (−0.71, 0.34); Fig. 1b], yet resulted in a significant decrease in error time from retrieval 1 (2.90 ± 6.09) to retrieval 2 (1.93 ± 4.71), [*Z *= −2.062, *P *= 0.039, *d *= 0.38, 95% CI (0.10, 2.75); Fig. 1c]. No significant differences were found between the nap and full‐night groups for either WPT or MTT performance during initial recall (retrieval 1), or the performance changes across the retention intervals (all *P *> 0.077). Statistically therefore the initial learning levels as well as the changes over day and night sleep were comparable (Fig. S6).

### Behavioural performance, sleep architecture and spindle activity

As a control (lacking an adaptation nap), we compared naps based on their chronological order, and found a shorter N2 onset latency (*P *= 0.030) and longer N2 duration (*P *= 0.017) for the second nap, whereas all other sleep variables remained unchanged (all *P *> 0.086; Table S1). Time spent in any of the sleep stages was unaffected by learning (all *P *> 0.063; Table 1). Yet, the procedural group showed a slightly longer total sleep time and higher sleep efficiency in the learning as compared with the control nap. Identical calculations for the full‐night study merely revealed an increase in N2 onset latency (*P *= 0.012) after learning for the declarative group. No significant changes were found for the procedural group (all *P *> 0.190; Table S2).

**Table 1:** Sleep architecture during control and learning naps for declarative and procedural learning

	Declarative WPT (*N *= 18)				Procedural MTT (*N *= 17)			
	Control nap	Learning nap	*t*‐value	*P*‐value	Control nap	Learning nap	*t*‐value	*P*‐value
Time in bed (min)	90.81 ± 1.09	91.44 ± 1.63	−1.52	0.146	90.91 ± 1.61	90.85 ± 0.90	0.13	0.898
Total sleep time (min)	71.83 ± 19.27	76.11 ± 12.18	−1.23	0.237	77.44 ± 8.38	81.82 ± 6.13	−2.22	0.041
Efficiency (%)	79.07 ± 20.93	83.20 ± 13.07	−1.07	0.300	85.23 ± 9.58	90.05 ± 6.50	−2.15	0.047
N2 latency (min)	14.19 ± 12.45	11.36 ± 6.50	1.28	0.219	12.00 ± 5.47	11.59 ± 5.44	0.27	0.793
N1 (%)	12.67 ± 7.98	14.34 ± 9.17	−0.77	0.451	24.56 ± 17.29	19.98 ± 11.28	1.02	0.322
N2 (%)	63.07 ± 16.38	57.38 ± 15.43	1.99	0.063	52.66 ± 17.77	54.42 ± 15.94	−0.37	0.718
SWS (%)	17.78 ± 17.54	24.04 ± 17.95	−1.69	0.110	16.74 ± 15.75	19.12 ± 15.79	−0.75	0.462
REM (%)	6.48 ± 9.40	4.24 ± 6.09	1.06	0.303	6.03 ± 7.09	6.49 ± 9.81	−0.20	0.844

REM, rapid eye movement; SWS, slow‐wave sleep; WPT, word‐pair task; MTT, mirror‐tracing task.

Note that none of the comparisons survive correction for multiple comparisons (*P *< 0.00625).

Next, we focused on associations between performance changes and sleep stages. For both WPT and MTT, no significant correlations were found between sleep stages and memory performance changes following Bonferroni correction for multiple comparisons using adjusted *P*‐value threshold (*P *= 0.00625). No significant correlations between performance changes and spindle or slow‐waves were found (all *P *> 0.124).

### Daytime nap versus full‐night sleep

Relative sleep architecture (in %) was compared between naps and full‐night sleep for control and learning conditions (Table 2). Sleep efficiency, N2 latency (in learning task only), N1%, and REM% all differed significantly between the nap and full‐night study (*P *≤ 0.005). In addition we contrasted sleep spindle characteristics (number, density, activity, frequency, and amplitude) between the nap and full‐night studies for both conditions, which revealed significant (1) lower spindle density, but (2) higher spindle activity, and (3) higher amplitude in the daytime nap (Table S3).

**Table 2:** Comparing sleep architecture during control and learning conditions between daytime naps and full‐night sleep

	Control condition				Learning condition			
	Nap (*N *= 35)	Night (*N *= 44)	*t*‐value	*P*‐value	Nap (*N *= 35)	Night (*N *= 44)	*t*‐value	*P*‐value
Time in bed (min)	90.86 ± 1.35	482.99 ± 34.99			91.16 ± 1.34	488.13 ± 26.88		
Total sleep time (min)	74.56 ± 15.06	454.51 ± 40.84			78.89 ± 10.01	451.66 ± 43.79		
Efficiency (%)	82.06 ± 16.49	94.10 ± 4.67	−4.19	<0.001^a^	86.53 ± 10.84	92.56 ± 7.60	−2.90	0.005^a^
N2 latency (min)	13.13 ± 9.64	19.01 ± 16.46	−1.87	0.065	11.47 ± 5.92	25.20 ± 21.76	−4.01	<0.001^a^
N1 (%)	18.44 ± 14.45	9.25 ± 4.71	3.62	0.001^a^	17.08 ± 10.49	9.55 ± 5.74	3.81	<0.001^a^
N2 (%)	58.02 ± 17.63	53.48 ± 8.06	1.41	0.166	55.94 ± 15.52	52.22 ± 11.20	1.19	.238
SWS (%)	17.28 ± 16.46	21.24 ± 5.75	−1.36	0.181	21.65 ± 16.87	21.97 ± 6.54	−0.11	0.916
REM (%)	6.26 ± 8.24	16.03 ± 5.11	−6.13	< 0.001^a^	5.33 ± 8.07	15.91 ± 6.49	−6.46	<0.001^a^

REM, rapid eye movement; SWS, slow‐wave sleep.

a Note that only *P *< 0.00625 is considered significant after Bonferroni correction for multiple comparisons.

Following earlier literature on the association between spindles and IQ (Bódizs *et al*., 2014; Fogel and Smith, 2011; Fogel *et al*., 2007; Schabus *et al*., 2006), we also investigated whether this association could be found in napping data. Yet, no significant associations were found between IQ and SpA during N2 (C3 and C4) when correcting for multiple comparisons (see Supplementary Material).

## Discussion

This study investigated the effects of napping on the consolidation of declarative and procedural information, and was performed as a multi‐centre study with the advantage to allow for increased generalizability. Surprisingly we did not find significant beneficial effects of a daytime nap on performance changes for either memory task, nor did we find associations between performance changes and sleep measures. Yet, a period of wakefulness trended towards reduced performance on a declarative memory task and resulted in a significant increase in error time on a procedural memory task. In other words, only in comparison to equivalent amounts of wakefulness were daytime naps beneficial. Using identical memory tasks, we compared these results with our previous full‐night study, for which declarative performance tended to increase and procedural performance significantly improved. Yet, given the huge variability in behavioural performance, a direct statistical comparison of performance across (day versus nighttime) sleep did not hold. For sleep architecture, neither our current daytime nap study nor our full‐night study showed associations of any sleep stage with declarative or procedural performance changes. Comparing sleep changes following learning (as compared with a control task) revealed a slight increase in sleep duration and efficiency after procedural mirror‐tracing, which may indicate that the task was cognitively more demanding than the control condition. At a more fine‐grained level no associations were found between sleep spindles or slow oscillations with procedural or declarative performance measures. The non‐significant associations between sleep architecture and declarative memory are somewhat unexpected given results from previous studies during daytime (Schabus *et al*., 2005; Tucker *et al*., 2006) and nighttime sleep (Plihal and Born, 1997; Wilhelm *et al*., 2008). Subtle study differences such as learning feedback, threshold learning before sleep (e.g. minimum 70% correct), and the (amount of) learning material could account for differences in the findings [e.g. word‐pair lists ranging from 40 (Wilhelm *et al*., 2008) to 168 word‐pairs (Gais *et al*., 2002)]; therefore making generalization between studies difficult.

Exploratory analyses contrasting nap and full‐night sleep architecture indicated several substantial differences besides mere sleep duration. Specifically we found more (relative) N1 sleep, less (relative) REM sleep and shorter N2 onset latency during naps. Furthermore, group comparisons regarding spindle characteristics showed robust differences in spindle density, activity, and amplitude. It can be speculated if differences of such kind may have direct consequences on aspects of memory consolidation. Especially, a practical absence of REM sleep and NREM–REM cycles as well as alterations in sleep spindles may be detrimental to certain kinds of memories. In addition, SWS amplitude and slope are significantly altered by prior time spent awake (Dijk *et al*., 1990), indicating that SWS itself likely differs in quality during naps. Finally, hormones such as acetylcholine, cortisol and norepinephrine are under circadian control (Roberts, 2000), and have been related to consolidation during sleep (Gais and Born, 2004). Altogether we believe that differences in such factors should be more carefully considered when discussing napping data and when generalizing results. Our current study and admittedly exploratory attempt to compare daytime naps with full‐night sleep is intended to highlight the manifold differences in sleep characteristics as well as performance outcomes, even if one uses completely identical memory tasks.

As in previous nighttime studies (Bódizs *et al*., 2014; Schabus *et al*., 2006), we also tested whether associations between IQ and sleep spindles can likewise be found in nap data. Surprisingly, the current study did not reveal associations of spindle activity and IQ, at least not after carefully correcting for multiple comparisons (Fig. S7).

Ideally this study should have included an adaptation sleep period as habitually performed for full‐night studies. Unfortunately this is rarely conducted for nap studies, although our data indicate that for a first nap N2 sleep onset latency is prolonged (by 3 min) and N2 percentage is reduced (by 9%; Table S1); whether such differences are relevant for sleep‐dependent memory consolidation is yet to be explored. As 94.7% of our participants were classified as moderate or intermediate chronotypes (D‐MEQ), an individual alignment to sleep times was considered negligible. A critical point to discuss and consider for the future is that our study may have been underpowered for finding some behavioural changes, especially for the WPT (see Supplementary Material for detailed power estimates). Yet, we would like to point out that the sample sizes of our subgroups (*n*_nap_ = 18; *n*_wake_ = 22) are comparable to other published studies using WPTs prior to sleep (Ellenbogen *et al*., 2009; *N *≤ 23; Fogel *et al*., 2007; *N *= 9; Gais *et al*., 2002; *N *= 16; Lahl *et al*., 2008; *N *= 26; Wilhelm *et al*., 2008, *N *= 15). We therefore believe that we as the whole field need to move to larger sample sizes and more carefully correct for multiple comparisons, especially when testing for associations of various memory parameters with numerous sleep architecture or sleep pattern (e.g. slow oscillation and spindle) measures.

Finally, our comparison of daytime and full‐night sleep would have benefitted from an additional control group staying awake across the full‐day (i.e. equivalent to nighttime sleep). Such an additional control group would have allowed to directly address performance changes across different retention intervals. Yet, for the full‐night study we previously did not include such a daytime wakefulness group as we believed that it adds uncontrollable confounds (i.e. time‐of‐day effects and difficulty to control interference during such extended retention intervals in the wake group) and is better compared with the same participants performing a control task before sleep. The nap study on the other hand included a wake group and was conducted under laboratory conditions in an attempt to keep interference to an absolute minimum. The contrast between daytime naps and full‐night sleep in our current study can therefore be considered as an ecological valid comparison of sleep‐dependent memory effects as they may occur in everyday life.

Given the current findings, we believe that the field would benefit from bigger multi‐centre studies, additional explicit comparisons between daytime and full‐night sleep, and using identical study material for a variety of memory domains. As discussed, daytime napping and full‐night sleep are different in many aspects, yet are often considered equivalent in currently published studies. It is to be confirmed whether this is justified.

## Conclusion

Daytime napping prevented deterioration of performance for procedural mirror‐tracing, with a similar trend observed for declarative word‐pair learning. Full‐night sleep led to an improvement of procedural memories over the retention period, whereas a trend towards improvement was observed for word‐pair learning. In addition, we found differences in spindles parameters between day and nighttime sleep, but no association of spindles, slow oscillations, or sleep stages with sleep‐dependent memory consolidation across a daytime nap. Altogether we believe that it is of importance to more carefully differentiate day and full‐night sleep, and to evaluate their relevance for memory consolidation.

## Author Contributions

CS, KH, JZ, and MS designed the study. KH, GK, MS, CS, and DM collected the data. FJVS, DPJH, and GG processed the data. FJVS and MS conducted statistical analyses. FJVS and MS drafted the manuscript. CS, KH, and MS supervised the project. All authors commented on and edited the manuscript drafts.

## Conflict of Interests

Research was funded by the Austrian Science Fund FWF (FWF P‐15370‐B02 & Doctoral College W1233‐G17). The authors report no conflict of interests.

## Supporting information


**Figure S1.** Word‐pair association study design.
**Figure S2.** Visualization of the MTT.
**Figure S3.** Behavioral performance for both WPT and MTT during control conditions.
**Figure S4.** Reports on sleepiness using the Stanford Sleepiness Scale (M ± SEM).
**Figure S5.** Procedural mirror‐tracing performance for nap and wake groups by task order (M ± SEM).
**Figure S6.** Behavioral performance for both WPT and MTT.
**Figure S7.** Association between intelligence scores and spindle activity during a daytime nap.
**Table S1.** Comparing sleep architecture of the first vs. second nap.
**Table S2.** Sleep architecture during control and learning nights for declarative and procedural learning.
**Table S3.** Spindle characteristics of daytime nap and full‐night sleep recordings in N2 sleep.
