# Supplementary material for: The effect of daytime napping and full‐night sleep on the consolidation of declarative and procedural information
Source: J Sleep Res. 2017 Dec 22;28(1):e12649. doi: 10.1111/jsr.12649 (PMC6378597; doi:10.1111/jsr.12649)
Supplement: Supplementary file 1 — Figure S1. Word‐pair association study design. Figure S2. Visualization of the MTT. Figure S3. Behavioral performance for both WPT and MTT during control conditions. Figure S4. Reports on sleepiness using the Stanford Sleepiness Scale (M ± SEM). Figure S5. Procedural mirror‐tracing performance for nap and wake groups by task order (M ± SEM). Figure S6. Behavioral performance for both WPT and MTT. Figure S7. Association between intelligence scores and spindle activity during a daytime nap. Table S1. Comparing sleep architecture of the first vs. second nap. Table S2. Sleep architecture during control and learning nights for declarative and procedural learning. Table S3. Spindle characteristics of daytime nap and full‐night sleep recordings in N2 sleep. [file JSR-28-na-s001.docx]

**Supplementary material**

**The effect of daytime napping and full-night sleep on the consolidation of declarative and procedural information.**

Frank J. van Schalkwijk^1^, Cornelia Sauter^2,3^, Kerstin Hoedlmoser^1^, Dominik P. J. Heib^1^, Gerhard Klösch^2^, Doris Moser^2^, Georg Gruber^4^, Peter Anderer^4^, Josef Zeitlhofer^2^, and Manuel Schabus^1,*^.

**
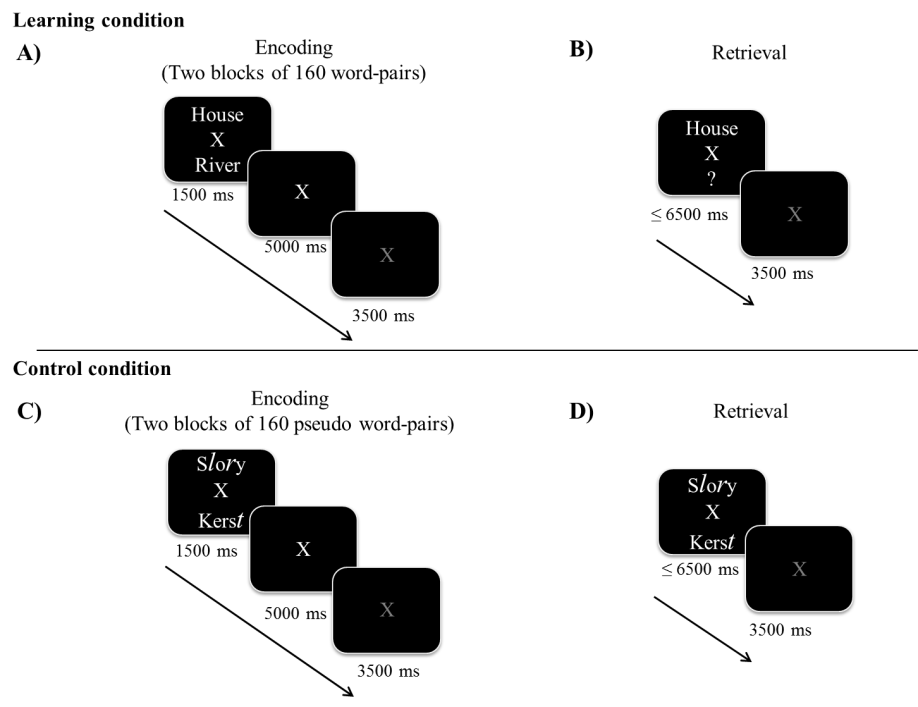
**

**Figure S1.** Word-pair association study design. (A) Participants had to associate word-pairs that were randomly displayed during two subsequent blocks. Word-pairs were displayed in white text on a black background for 1500 ms. Word-pairs were directly followed by a white central fixation cross for 5000 ms. A gray central fixation cross separated trials for 3500 ms. (B) Retrieval only showed the first word of the learned word-pair; requiring participants to verbally report the associated word. (C-D) Encoding and retrieval conditions for the control condition. Participants were shown pseudo word-pairs for which they had to count and report the number of alternatively displayed letters (italic and larger font size). Hence, the control condition was almost identical to the leaning task but excluded the memory aspect.


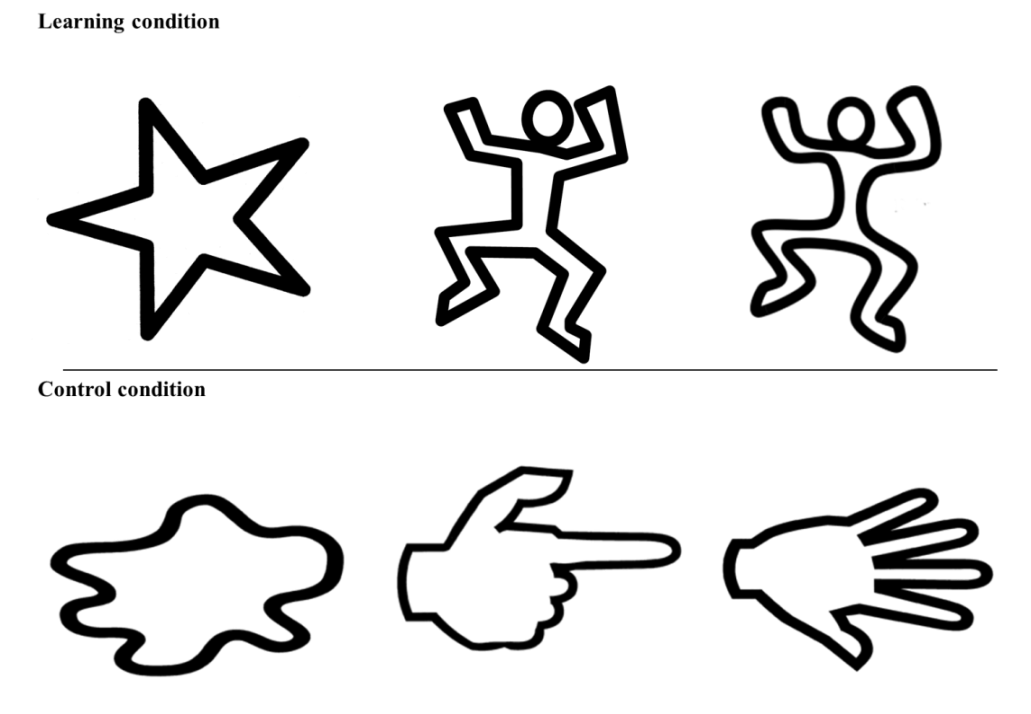


**Figure S2.** Visualization of the MTT. Each session started with two dummy stimuli serving as training (left stimuli) and were not used in the analyses. The 12 relevant stimuli were presented twice in a blocked and randomized order. A similar paradigm was used for both learning (top) and control (bottom) conditions; only differing in the general shape of stimuli shown.


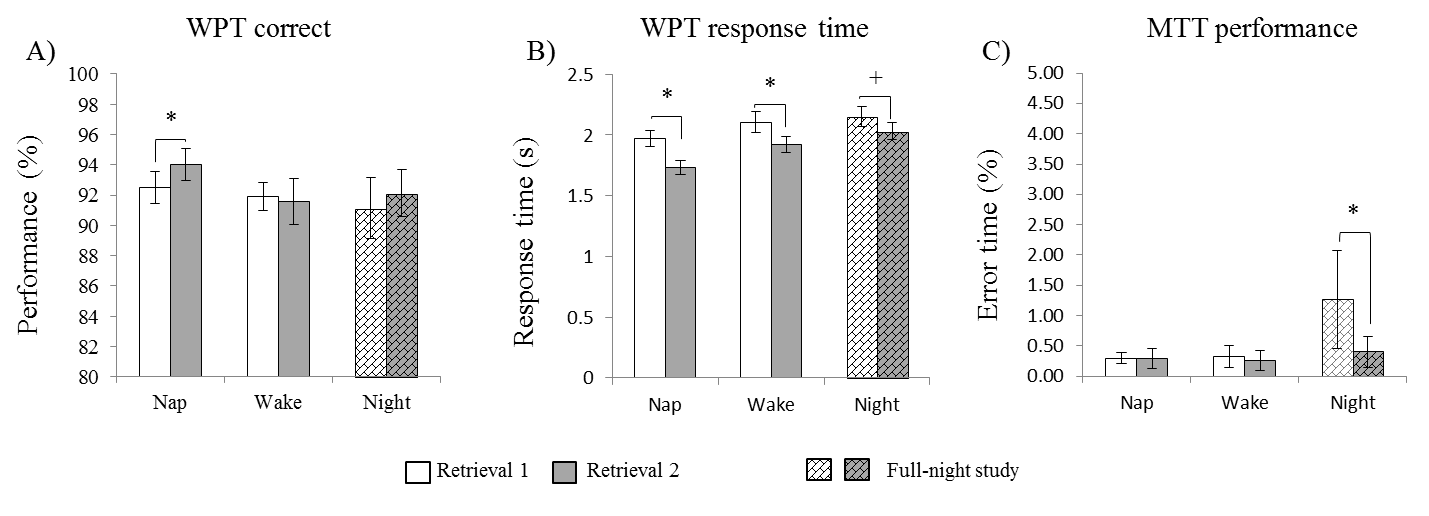


**Figure S3.** Behavioral performance for both WPT and MTT during control conditions. Plots illustrate mean ± standard error. Error bars illustrate between-subject variability. Performance on the WPT control task was evaluated for accuracy (A), which showed an increase in the nap group only. In addition, average response time (B) significantly improved for the nap and wake group, whereas the full-night sleep group showed a trending decrease. For the MTT task (C), no performance changes were observed for the nap and wake group, whereas a significant decrease was observed for the full-night sleep group. * *p* < .05, + *p* < .10.


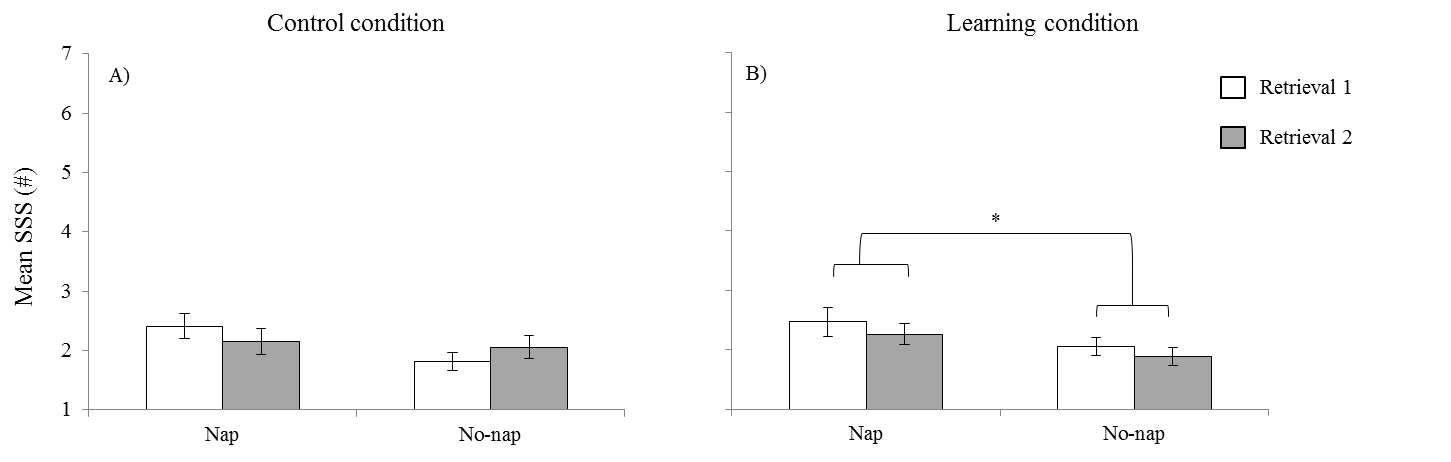


**Figure S4. Reports on sleepiness using the Stanford Sleepiness Scale (*M* ± *SEM*).** Fatigue was reported by participants for the full duration of the paradigm on a scale from 1-7 (1 = “Feeling active, vital, alert, or wide awake”; 2 = “Functioning at high levels but not at peak; able to concentrate”; 3 = “Awake but relaxed; responsive but not fully alert”; 4 = “Somewhat foggy, let down”; 5 = “Foggy; losing interest in remaining awake; slowed down”; 6 = “Sleepy, woozy, fighting sleep; prefer to lie down”; 7 = “No longer fighting sleep, sleep onset soon; having dreamlike thoughts”).


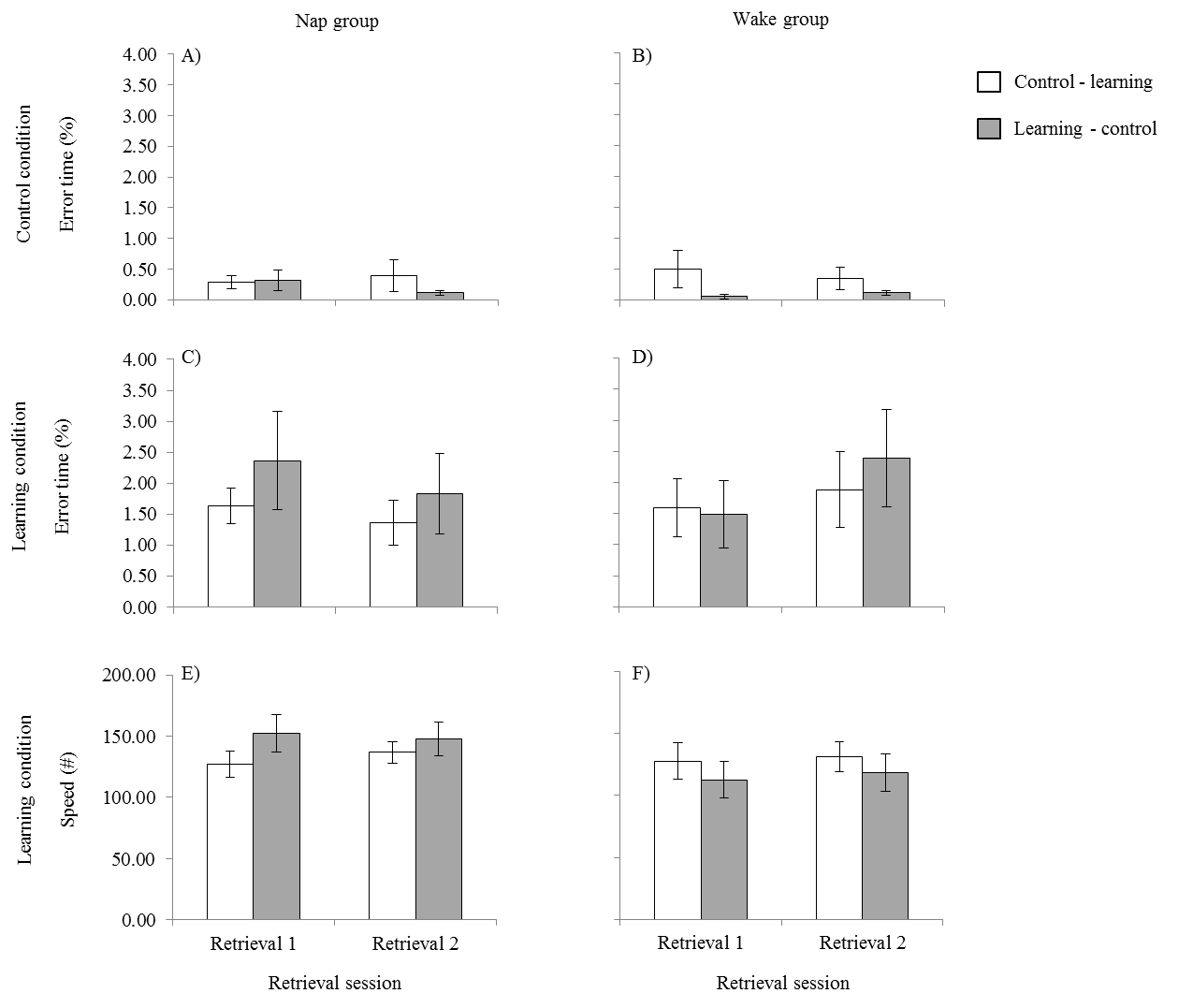


**Figure S5. Procedural mirror-tracing performance for nap and wake groups by task order (*M* ± *SEM*)**. Plots are sorted by condition order, that is control before learning vs. learning before control task and illustrate behavioral performance on MTT error time during control (A-B) and learning (C-D) conditions, as well as speed during the learning condition (E-F). Note that no significant differences are found in the retrieval 1 to retrieval 2 performance changes when directly comparing these two groups (all *p*s > .199).

**
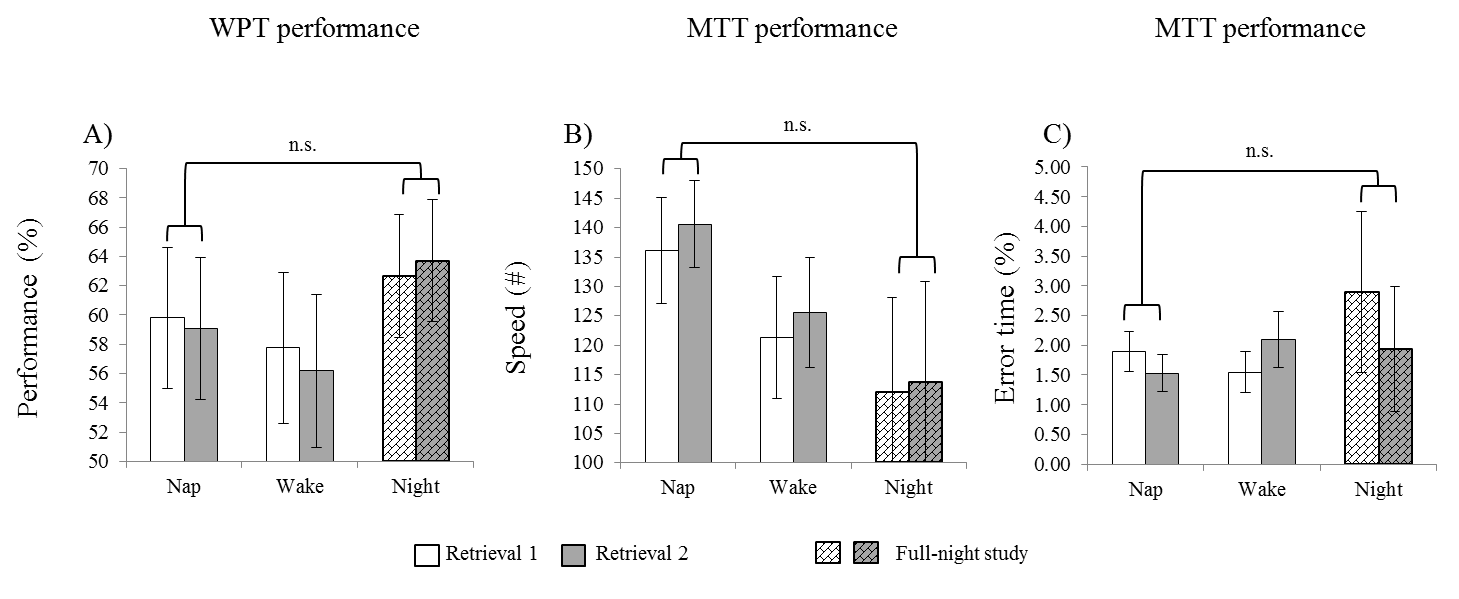
**

**Figure S6.** Behavioral performance for both WPT and MTT. Error bars illustrate between-subject variability (*M* ± *SEM*). For the WPT (A), no group differences were observed for performance during retrieval 1, *t*(40) = -.443, *p* = .660, *d* = -.14, 95% CI [-.77, .49], nor was there a difference in performance change, *t*(40) = -1.804, *p* = .079, *d* = -.56, 95% CI [-1.2, .08]. For the MTT task (B-C), no group differences were observed for speed (B) during retrieval 1, *t*(20.90) = 1.306, *p* = .206, *d* = .43, 95% CI [-.25, 1.11], nor for performance changes, *t*(25.31) = .623, *p* = .539, *d* = .21, 95% CI [-.47, .88]. Similarly, no group differences were observed for error time (C) during retrieval 1, *Z* = -1.21, *p* = .228, *d* = -.28, 95% CI [-.93, .37], nor for performance changes, *Z* = -.122, *p* = .903, *d* = .36, 95% CI [-.29, 1.01].

| **Table S1.** Comparing sleep architecture of the first vs. second nap. | | | | |
| --- | --- | --- | --- | --- |
|  | First nap | Second nap | *t*  value | *p*  value |
| Time in bed (min) | 91.06 ± 1.70 | 90.96 ± .88 | .32 | .751 |
| Total sleep time (min) | 74.91 ± 15.10 | 78.53 ± 10.10 | -1.77 | .086 |
| Efficiency (%) | 82.26 ± 16.41 | 86.33 ± 11.04 | -1.80 | .080 |
| N2 onset latency (min) | 13.77 ± 9.58 | 10.83 ± 5.75 | 2.27 | .030 |
| N1 (%) | 20.48 ± 14.62 | 17.48 ± 17.95 | 1.00 | .324 |
| N2 (%) | 54.82 ± 16.74 | 64.22 ± 21.31 | -2.50 | .017 |
| SWS (%) | 20.04 ± 16.28 | 23.92 ± 28.67 | -.83 | .415 |
| REM (%) | 4.66 ± 6.96 | 7.10 ± 9.36 | -1.56 | .128 |
| Note that none of the comparisons survive correction for multiple comparisons (p < .00625). | | | | |

| **Table S2.** Sleep architecture during control and learning nights for declarative and procedural learning. | | | | | | | | | | |
| --- | --- | --- | --- | --- | --- | --- | --- | --- | --- | --- |
|  | | Declarative WPT (*N* = 24) | | | | | Procedural MTT (*N* = 20) | | | |
|  | Control  condition | | Learning condition | *t*  value | | *p*  value | Control  condition | Learning condition | *t*  value | *p*  value |
| Time in bed (min) | 479.27 ± 26.91 | | 491.25 ± 15.78 | -1.81 | .083 | | 487.45 ± 43.08 | 484.38 ± 36.15 | .43 | .675 |
| Total sleep time (min) | 443.44 ± 34.58 | | 440.96 ± 45.73 | .29 | .772 | | 467.80 ± 44.56 | 464.50 ± 38.59 | .50 | .626 |
| Efficiency (%) | 92.57 ± 5.56 | | 89.82 ± 9.41 | 2.07 | .050 | | 95.93 ± 2.33 | 95.85 ± 1.66 | .18 | .863 |
| N2 latency (min) | 22.67 ± 19.87 | | 31.94 ± 25.95 | -2.71 | .012 | | 14.63 ± 9.89 | 17.13 ± 11.41 | -.92 | .369 |
| N1 (%) | 10.29 ± 4.96 | | 10.76 ± 7.01 | -.45 | .658 | | 8.00 ± 4.17 | 8.11 ± 3.34 | -.11 | .914 |
| N2 (%) | 51.56 ± 8.12 | | 50.59 ± 12.91 | .46 | .650 | | 55.79 ± 7.54 | 54.19 ± 8.63 | 1.36 | .190 |
| SWS (%) | 22.39 ± 5.88 | | 22.83 ± 7.27 | -.58 | .570 | | 19.87 ± 5.42 | 20.94 ± 5.56 | -.81 | .431 |
| REM (%) | 15.76 ± 5.31 | | 15.52 ± 7.61 | .20 | .847 | | 16.35 ± 4.97 | 16.38 ± 4.98 | -.02 | .983 |
| Note that none of the comparisons survive correction for multiple comparisons (p < .00625). | | | | | | | | | | |

| **Table S3.** Spindle characteristics of daytime nap and full-night sleep recordings in N2 sleep. | | | | | | |
| --- | --- | --- | --- | --- | --- | --- |
|  |  |  | **Control condition** | | **Learning condition** | |
|  | *Study* | *Electrode* | *Slow spindles* | *Fast spindles* | *Slow spindles* | *Fast spindles* |
| Spindle number | Nap study | C3 | 54 ± 46 | 165 ± 153 | 56 ± 52 | 151 ± 119 |
|  |  | C4 | 51 ± 47 | 156 ± 146 | 51 ± 49 | 141 ± 116 |
|  | Full-night study | C3 | 519 ± 364 | 1343 ± 621 | 482 ± 365 | 1331 ± 634 |
|  |  | C4 | 511 ± 359 | 1339 ± 591 | 474 ± 361 | 1268 ± 671 |
| Spindle percentage | Nap study | C3 | 24% ± 15% | 76% ± 15% | 25% ± 14% | 75% ± 14% |
|  |  | C4 | 23% ± 14% | 77% ± 14% | 26% ± 14% | 74% ± 14% |
|  | Full-night study | C3 | 27% ± 13% | 73% ± 13% | 26% ± 14% | 74% ± 14% |
|  |  | C4 | 28% ± 14% | 72% ± 14% | 28% ± 17% | 72% ± 17% |
| *p*-values | | C3 | .323 | .323 | .620 | .620 |
|  |  | C4 | .167 | .167 | .559 | .559 |
| Spindle density | Nap study | C3 | 1.19 ± 0.98 | 3.45 ± 2.49 | 1.22 ± 1.02 | 3.26 ± 2.14 |
|  |  | C4 | 1.12 ± 1.01 | 3.27 ± 2.42 | 1.12 ± 0.99 | 3.08 ± 2.07 |
|  | Full-night study | C3 | 2.10 ± 1.34 | 5.50 ± 2.32 | 1.99 ± 1.27 | 5.62 ± 2.31 |
|  |  | C4 | 2.08 ± 1.33 | 5.48 ± 2.18 | 1.93 ± 1.24 | 5.33 ± 2.45 |
| *p*-values | | C3 | .001^+^ | < .001* | .006 | < .001* |
|  |  | C4 | .001^+^ | < .001* | .003 | < .001* |
| Spindle activity (SpA) | Nap study | C3 | 20.62 ± 4.08 | 21.39 ± 4.45 | 21.56 ± 4.76 | 21.23 ± 4.42 |
|  |  | C4 | 20.38 ± 4.14 | 21.02 ± 5.15 | 20.83 ± 4.04 | 21.20 ± 4.09 |
|  | Full-night study | C3 | 15.74 ± 2.31 | 16.90 ± 3.27 | 15.82 ± 2.48 | 17.27 ± 3.38 |
|  |  | C4 | 15.83 ± 2.43 | 17.17 ± 3.19 | 15.87 ± 2.48 | 17.03 ± 3.38 |
| *p*-values | | C3 | < .001* | < .001* | < .001* | < .001* |
|  |  | C4 | < .001* | < .001* | < .001* | < .001* |
| Spindle frequency | Nap study | C3 | 12.63 ± 0.11 | 13.71 ± 0.17 | 12.63 ± 0.12 | 13.70 ± 0.19 |
|  |  | C4 | 12.58 ± 0.14 | 13.68 ± 0.20 | 12.61 ± 0.13 | 13.70 ± 0.16 |
|  | Full-night study | C3 | 12.61 ± 0.08 | 13.66 ± 0.23 | 12.61 ± 0.08 | 13.68 ± 0.22 |
|  |  | C4 | 12.61 ± 0.09 | 13.66 ± 0.23 | 12.59 ± 0.10 | 13.67 ± 0.23 |
| *p*-values | | C3 | .262 | .202 | .312 | .577 |
|  |  | C4 | .290 | .650 | .524 | .526 |
| Spindle amplitude | Nap study | C3 | 22.62 ± 3.23 | 22.87 ± 3.95 | 22.92 ± 2.98 | 22.61 ± 3.77 |
|  |  | C4 | 22.02 ± 3.18 | 22.64 ± 3.87 | 22.11 ± 2.89 | 22.57 ± 3.38 |
|  | Full-night study | C3 | 17.35 ± 2.14 | 18.11 ± 2.78 | 17.51 ± 2.38 | 18.45 ± 3.09 |
|  |  | C4 | 17.60 ± 2.29 | 18.39 ± 2.80 | 17.57 ± 2.46 | 18.43 ± 2.96 |
| *p*-values | | C3 | < .001* | < .001* | < .001* | < .001* |
|  |  | C4 | < .001* | < .001* | < .001* | < .001* |
| *p*-values <.001 are considered significant given multiple comparisons (result from independent-sample *t*-tests). For obvious reasons no t-tests have been performed for absolute spindle number in the nap vs. the full-night study. | | | | | | |

**Correlations between spindles and IQ**

Across the nap conditions a positive correlation between IQ and mean slow SpA was found during N2 on channel C4 (*r* = .31, *n* = 67, *p* = .01). As prior studies indicated potential effects by gender (Ujma et al., 2014, Bódizs et al., 2014) an additional gender split was performed. While for male participants a positive correlation between IQ and mean SpA was found for both slow (*r* = .375, *n* = 37, *p* = .022; Fig. S7) and fast spindles (*r* = .327, *n* = 38, *p* = .045), female participants only revealed a positive association of IQ and mean SpA for slow (*r* = .383, *n* = 30, *p* = .037; Fig. S7) but not fast spindles (*r* = .205, *n* = 31, *p* = .268). However, after Bonferroni correction for multiple comparisons (spindle type, channel, and gender), the previously reported effects were not significant in light of the corrected minimum p-value threshold (*p* = .00625).

**
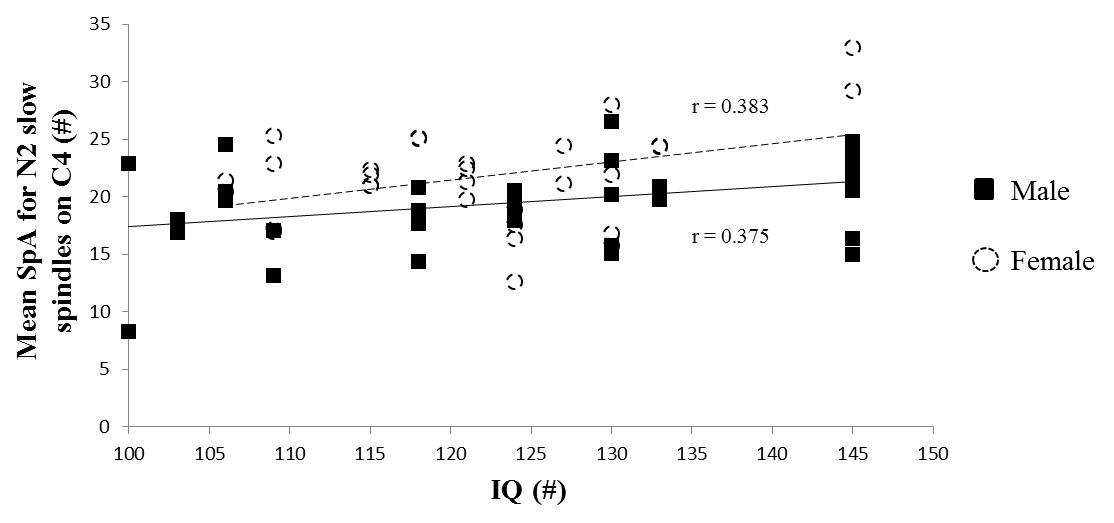
**

**Figure S7.** Association between intelligence scores and spindle activity during a daytime nap. Correlations between IQ and mean slow SpA on electrode C4 for a nap during both control and learning condition. A gender split showed a positive correlation between IQ and SpA for slow-spindles on C4 for male and female participants. However, these effects do not survive correction for multiple comparisons (Bonferroni; *p* < .00625).

**Power estimates**

For the word-pair association task we retrieved statistical values from the original paper by Plihal and Born (1997). They reported significant overnight improvement on the amount of recalled word-pairs, with the strongest effect for early sleep (*F*(1, 9) = 33.19, *p* < 0.001) as compared to late sleep *F*(1, 9) = 5.04, *p* < 0.05). The weakest effect yielded a Cohen’s d value of 1.00. Using half the effect size (.50) and 95% confidence level, our sample (*n*_nap_ = 18, *n*_wake_ = 22) yielded a power estimate of 0.516 (51.6%) for within-subject effects and 0.335 (33.5%) for between-subject effects.

For power estimates for the MTT, we refer to the original study by Plihal and Born (1997) who showed within-subject effects on mirror-tracing error time from early and late sleep retention in 10 subjects. Using the weakest reported effect (*F*(1,7) = 7.83, *p* < .005) yielded a Cohen’s *d* value of 1.25. Using half the effect size (0.625) and 95% confidence level, our lowest group sample (*N* = 17) and significance level of .05 yields a power estimate of .677 (67.7%) for the MTT for within-subject effects and 0.424 (42.4%) for between-subject effects. Similarly, the study by Schonauer et al. (2015) showed a within-subjects effect on mirror-tracing error time between sleep and sleep deprivation conditions, *F*(1,20) = 22.2, *p* < .001, which yielded a Cohen’s d value of 1.45. Using half the effect size (0.725) and 95% confidence level, our lowest group sample (*N* = 17) and significance level of .05 yields a power estimate of 0.801 (80.1%) for within-subject effects and 0.560 (56%) for between-subject effects; this can be considered adequate for within-subject effects in MTT behavioral performance.
